# Extra-Adrenal Glucocorticoid Synthesis in the Intestinal Mucosa: Between Immune Homeostasis and Immune Escape

**DOI:** 10.3389/fimmu.2019.01438

**Published:** 2019-06-25

**Authors:** Asma Ahmed, Christian Schmidt, Thomas Brunner

**Affiliations:** ^1^Biochemical Pharmacology, Department of Biology, University of Konstanz, Konstanz, Germany; ^2^Department of Pharmacology, Faculty of Medicine, University of Khartoum, Khartoum, Sudan

**Keywords:** glucocorticoids, intestinal mucosa, intestinal immune homeostasis, inflammatory bowel disease, colorectal cancer, liver receptor homolog-1, tumor necrosis factor

## Abstract

Glucocorticoids (GCs) are steroid hormones predominantly produced in the adrenal glands in response to physiological cues and stress. Adrenal GCs mediate potent anti-inflammatory and immunosuppressive functions. Accumulating evidence in the past two decades has demonstrated other extra-adrenal organs and tissues capable of synthesizing GCs. This review discusses the role and regulation of GC synthesis in the intestinal epithelium in the regulation of normal immune homeostasis, inflammatory diseases of the intestinal mucosa, and the development of intestinal tumors.

## General Aspects of Glucocorticoids

### Glucocorticoids

Glucocorticoids (GCs) are immunoregulatory hormones synthesized in the adrenal cortex and secreted into the blood in a circadian mode under physiological and stress conditions (1). GCs regulate fundamental body functions in mammals including control of cell growth, development, metabolic homeostasis, cognition, mental health, immune homeostasis, and apoptosis (2–5). In the 1940s GCs were discovered as extracts of the adrenal cortex. This was followed by the isolation of adrenocorticotropic hormone (ACTH) from pituitary gland extracts. In 1950, Kendall, Reichstein, and Hench were awarded the Nobel Prize in Physiology and Medicine for their pioneering work in describing that GCs had a powerful anti-inflammatory effect in the treatment of rheumatoid arthritis (6, 7). Since the 1950s, and owing to their strong anti-inflammatory and immunosuppressive activities, GCs have been widely used for the treatment of inflammatory disorders and autoimmune diseases, such as asthma, rheumatoid arthritis, dermatitis, inflammatory bowel disease (IBD), sepsis, lupus erythematosus, and multiple sclerosis (7–11). GCs are also used as immunosuppressive drugs following organ transplantation and in the treatment of leukemia (11, 12).

Immunological, environmental, and emotional stress induces the release of GCs to mediate immunoregulatory activities, mostly immunosuppressive, on distant tissues and cells, in particular in immune cells (4). For example, GCs have an immunosuppressive activity on T cell-mediated immune responses (13) and this is why they are frequently used for the treatments of T cell-mediated immunopathologies.

**Figure 1:**
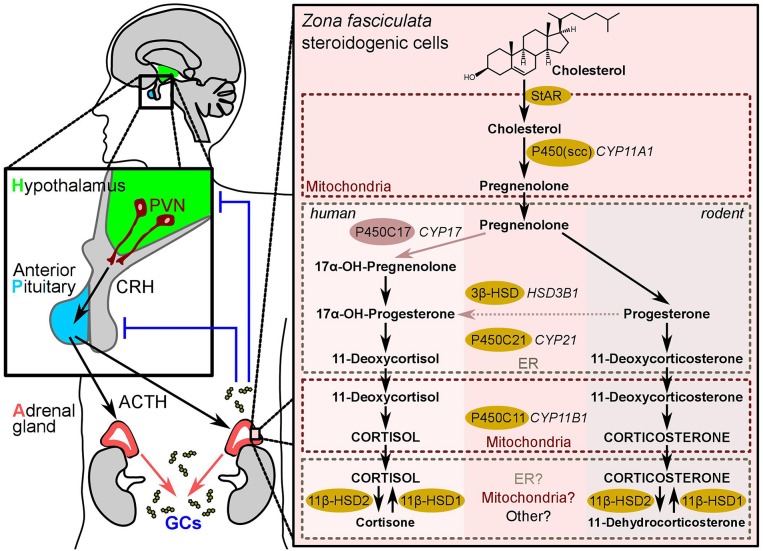
The HPA-axis and adrenal glucocorticoid synthesis. The Hypothalamus-pituitary-adrenal-axis (also known as “stress axis”) represents the sequence of endocrine events between the hypothalamus (green), the anterior pituitary gland (blue), and the cortex of the adrenal gland (red). Corticotropin-releasing hormone (CRH) secreted from the paraventricular neurons (PVNs) of the hypothalamus stimulates adrenocorticotropic hormone (ACTH, corticotropin) release from the anterior pituitary, which consequently stimulates the production of glucocorticoids in the steroidogenic cells of the *zona fasciculata* in the adrenal cortex. Blue lines indicate negative feedback. The right-hand panel shows the biochemical reactions leading to glucocorticoid-synthesis in humans and in rodents. The synthesizing enzymes are shown in yellow (and light-red for the human CYP17). The (so far known) subcellular localization of the steroidogenic enzymes in the mitochondria or the ER is highlighted by dotted-line boxes.

The synthesis of adrenal GCs is regulated by the hypothalamic-pituitary-adrenal (HPA) axis (Figure 1), and controlled by the main circadian oscillator located in the suprachiasmatic nucleus (SCN) of the hypothalamus (1). Basal and stress-inputs to the hypothalamus promote the release of corticotropin-releasing hormone (CRH) from neurosecretory cells of the paraventricular nucleus (PVN), which stimulates the synthesis and secretion of ACTH (corticotropin) from the anterior pituitary gland. ACTH in turn promotes the production and secretion of GCs (cortisol in humans and corticosterone in rodents) from the adrenal cortex (14) (Figure 1). Afterwards, GCs target the hypothalamus and the anterior pituitary to inhibit the release of CRH and ACTH in a negative feedback loop (Figure 1). GCs act on almost all types of cells in the body to maintain homeostasis both, in response to normal diurnal changes in metabolism and in response to stress (2, 3). Noteworthy, inflammatory cytokines including interleukin-1 beta (IL-1β), IL-6, and tumor necrosis factor alpha (TNF) were also reported to stimulate the release of ACTH and CRH further indicating the bidirectional communication between immune and neuroendocrine systems (15).

### Adrenal GC Synthesis

Adrenal GCs are synthesized and released by the *zona fasciculata* of the adrenal cortex in a circadian manner, as well as in response to environmental and immunological stress (16). GCs are synthesized from the precursor cholesterol and the synthesis is regulated by the transcriptional control of the steroidogenic enzymes that involve cytochrome P450 (CYP) oxidative enzymes and hydroxysteroid dehydrogenase (HSD) enzymes (1) (Figure 1). The first step in steroidogenesis takes place within mitochondria, where cholesterol is transported from the outer to the inner mitochondrial membrane by the steroidogenic acute regulatory protein (StAR) (17). The first and rate-limiting step in steroid synthesis is the conversion of cholesterol to pregnenolone by the action of side-chain cleavage enzyme, P450scc, encoded by the *CYP11A1* gene (Figure 1). Thus, it is the expression of P450scc that renders a cell steroidogenic, i.e., able to synthesize steroids *de novo*. Supporting this notion, mice with a deletion of the *Cyp11a1* gene suffer from steroid deficiency (17–19). In humans, once pregnenolone is produced from cholesterol, it undergoes 17α-hydroxylation by P450c17 (CYP17) to yield 17α-hydroxypregnenolone. Next, pregnenolone is converted to progesterone by 3β-HSD (20). Afterwards, 21-hydroxylase (CYP21) converts progesterone into 11-deoxycortisol (humans) or 11-deoxycorticosterone (rodents), then 11β-hydroxylase encoded by the *CYP11B1* gene catalyzes the last hydroxylation step in the GC synthesis. The last step comprises the conversion of 11-deoxycortisol to cortisol in humans, and 11-deoxycorticosterone to corticosterone in rodents, since the rodent adrenals lack CYP17 enzyme (20, 21) (Figure 1).

Several factors have been shown to contribute to and modify the cellular and organismal responses to GCs. Notably, most of the secreted cortisol in the blood (~90%) is bound to proteins (corticosteroid-binding globulins and albumin). This binding regulates the general availability of GCs to tissues and/or direct the delivery of hormones to specific sites (22–24).

It is known that the presence of an 11β-hydroxyl group is essential for the anti-inflammatory and immunosuppressive effects of GCs and for the sodium-retaining effects of the mineralocorticoids (MCs). Therefore, it has been shown that the isoenzymes of 11β-hydroxysteroid dehydrogenase (11β-HSD) critically regulate the conversion between the active and the inactive form of a steroid in target cells. 11β-HSD2 catalyzes the conversion of cortisol, the biologically active form, to the inactive cortisone, whereas 11β-HSD1 converts cortisone to cortisol. Thus, 11β-HSD1, which is expressed in a wide range of tissues and predominantly in the liver, facilitates GC hormone actions whereas the major role of 11β-HSD2 is to prevent cortisol from gaining access to high-affinity MC receptors. Therefore, 11β-HSD2 is predominantly expressed in the MC responsive cells of the kidney and other MC target tissues such as the colon (11).

Adrenal GC synthesis is regulated by the orphan nuclear receptor (NR) steroidogenic factor 1 (SF-1), encoded by the *NR5A1* (nuclear receptor subfamily 5, group A, member 1) gene. SF-1 plays a key role in the development and function of steroidogenic tissues, and has emerged as a key regulator of endocrine function within the hypothalamic-pituitary-gonadal axis and adrenal cortex, and as an essential factor in sex differentiation. SF-1 was first identified as an essential regulator of endocrine development and function, including steroid hormone biosynthesis, via induction of the expression of steroidogenic enzymes, including CYP11A1, CYP17, CYP21, CYP11B1, and 3β-HSD. Similarly, SF-1 has been reported to regulate the expression of StAR as well as the ACTH receptor (25, 26).

### Glucocorticoid Receptor Activation

GCs act via genomic (transcriptional) and non-genomic (transcription-independent) mechanisms (27). Most cellular actions of GCs are primarily mediated via binding to their cognate intracellular receptor, the classic glucocorticoid receptor (GR) protein, GRα. GR is a ligand-regulated transcription factor (TF) that belongs to the NR subclass 3C and is therefore known as NR3C1 (nuclear receptor subfamily 3, group C, member 1). In line with the pleiotropic actions of GCs, GR is expressed in nearly every cell of the body and is essential for life after birth. Alternative mRNA splicing results in a second GR isoform, GRβ. GRβ does not bind to GC agonists, resides constitutively in the nucleus, and is inactive by itself. However, when co-expressed with GRα, GRβ functions as a dominant negative inhibitor of GRα (2, 28–31).

The GRα shares common structural and functional domains with other NRs. These domains include an N-terminal ligand-independent transactivation domain, also called activation function 1 (AF-1), which is responsible for the transcription activation, a highly conserved DNA-binding domain (DBD) that is important for GR homodimerization and DNA-binding specificity, a C-terminal ligand-binding domain (LBD) that contains the ligand-binding site and a second ligand-dependent transactivation domain (AF-2), and a flexible hinge region separating the DBD and the LBD (32–34). In addition to the known dimerization function of the DBD, *in vivo* evidence has shown that LBD mutation severely compromised GR dimerization, whereas no correlation between oligomerization state, DNA binding, and transcriptional activity could be established (35). These data clearly indicate that multiple domains are involved in GR dimerization.

In the absence of ligand, the GRα is sequestered predominantly in the cytoplasm as an inactive multi-protein complex formed by chaperonic molecules, including heat shock proteins Hsp90, Hsp70, Hsp23, and immunophillins p59 and calreticulin (28, 29, 36). These proteins maintain the receptor in a conformation that is transcriptionally inactive, but favors high affinity ligand binding (2). Binding of endogenous or synthetic GCs to the LBD of GRα induces receptor conformational change leading to the dissociation of the multi-protein complex and allows the translocation of the GC/GR complex to the nucleus where it regulates gene transcription (21). Upon translocation to the nucleus, the GRα binds DNA sequences, known as GC response elements (GREs), to positively or negatively regulate gene transcription by direct DNA-binding or by interaction with other proteins (3, 37).

In addition to the transcription activation, the GR represses a wide variety of genes. This repression function is mediated by negative GREs (nGRE) in the promoter regions of target genes. nGREs contribute to the negative feedback of HPA axis, bone, and skin function, inflammation, angiogenesis, and lactation. Moreover, GR inhibits glycoprotein hormone promoter, which is positively regulated by the cyclic adenosine monophosphate (cAMP) response element binding protein (CREB) and contains binding sites for CREB and GR. Upon DNA binding, GR inhibits transcription activation directly by preventing CREB binding (28, 38, 39).

Accumulating evidence suggests that GCs can act via non-genomic mechanisms to elicit more rapid cellular responses (within seconds to minutes) that do not require nuclear GR-mediated changes in gene expression. The non-genomic effects of GCs are considered to be mediated through binding to membrane-bound GR, binding to cytosolic GR, or by interactions with cellular membranes (30, 40, 41). Bartholome et al. showed that membrane GRs are expressed in human monocytes and B cells (42). Additionally, they monitored a strong positive correlation between the frequency of membrane GR-positive monocytes and various parameters of disease activity in patients with rheumatoid arthritis. This observation prompted the authors to suggest that immunostimulation induces the expression of membrane GR in immune cells such as monocytes that in turn triggers rapid signal cascades leading to a significantly higher percentage of cells to undergo GC-induced apoptosis to limit excessive immune reaction (42). GCs can also bind to their cytosolic GR to induce rapid non-genomic effects resulting in interactions with signaling pathways. For example, GCs were shown to activate endothelial nitric oxide synthase in a non-genomic manner and mediated by stimulated phosphatidylinositol 3-kinase and protein kinase Akt phosphorylation (41, 43). High concentrations of GCs have been shown to induce quantitative increase in the intercalation of GC molecules in the membrane, influencing the membrane fluidity, membrane associated proteins and cation uptake, as measured by the reduction of cation transport ATPase activity (44, 45).

### Glucocorticoid Functions

#### Anti-inflammatory Functions of GCs

**Figure 2:**
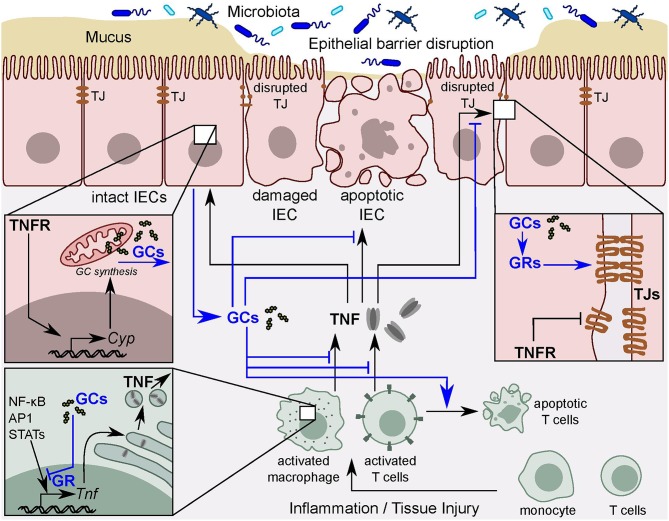
TNF and intestinal GC synthesis. Intestinal epithelial barrier disruption leads to permeability defects and the subsequent interaction of intestinal immune cells with the luminal contents. Activated immune cells release pro-inflammatory cytokines, such as TNF. In turn, TNF results in tight junction (TJ) disruption and intestinal epithelial cell (IEC) apoptosis and thereby exacerbates local inflammation. TNF also directly stimulates IECs to synthesize and release immunoregulatory glucocorticoids (GCs) to counter-balance excessive tissue damage. GCs act via the glucocorticoid receptor (GR) to inhibit TNF-mediated tissue damage in a negative feedback loop. The GR also inhibits pro-inflammatory transcription factors, including NF-κB, AP-1, and STATs leading to the resolution of the inflammation.

Upon tissue injury, irritants or pathogen invasion, immune cells of the innate, and adaptive immune systems are activated and recruited to the site of inflammation (12, 46). Immune cells activation and recruitment is mediated by cytokines and chemokines, which are regulated by inflammatory TFs, including the nuclear factor 'kappa-light-chain-enhancer' of activated B-cells (NF-κB), signal transducer and activator of transcription proteins (STATs) and activator protein 1 (AP-1) (46). These TFs are crucial regulators of a variety of cellular functions, including cell survival, proliferation, differentiation, and apoptosis (47–50). In the presence of pro-inflammatory stimuli, these TFs trigger activation of pro-inflammatory cytokines, such as TNF, IL-1β, and IL-6 among others, to induce inflammation and promote cell survival (51). GR induces anti-inflammatory activities by direct interaction with other TFs, including NF-κB (51), STAT3 (52), STAT5, and AP-1, leading to their inhibition, thus repressing the expression of pro-inflammatory genes and thereby promoting the resolution of inflammation (29) (Figure 2). Since this interaction does not require DNA binding, the term tethering GRE is often used to describe these elements. Interestingly, tethering GREs do not contain DNA binding sites for GRs, but instead contain binding sites for other DNA-bound regulators, including NF-κB and AP-1, that recruit GRs (28, 53).

GCs also induce proteins with anti-inflammatory activities, including glucocorticoid-induced leucine zipper (GILZ), resulting in the inhibition of the mitogen-activated protein kinase (MAPK) pathway (27). MAPK activation is associated with cell proliferation, differentiation, migration, senescence and apoptosis [reviewed in (54)]. Another mechanism, by which GILZ dictates its anti-inflammatory function, is via inhibition of NF-κB and AP-1 activities (27, 55).

#### Immunosuppressive and Metabolic Functions of GCs

GCs have powerful immunosuppressive activities mediated by acting on almost all types of cells, in particular on immune cells (33). GCs induce apoptosis in a variety of immune cells, including developing thymocytes as well as circulating and tissue-resident T cells, mediated by the pro-apoptotic proteins Puma and Bim (56–58). GCs also promote dendritic cell (DC) apoptosis (29). Additionally, GCs favor the expansion of immunosuppressive regulatory T cells (Tregs) by upregulating the expression of FoxP3, the master regulator of Tregs (59, 60). Moreover, GCs promote the shift from T helper 1 (Th1) to Th2 immune responses by differentially regulating apoptosis of Th1 and Th2 cells (13, 61–63).

GCs also control the function of innate immune cells, including monocytes and macrophages, in order to regulate tissue homeostasis. GCs have been shown to induce the differentiation and promote the survival of anti-inflammatory (M2) macrophages, evident by the induced expression of the immunomodulatory cytokine IL-10. This effect is mediated by prolonged activation of the MAPK pathway resulting in inhibition of caspase activities, and expression of anti-apoptotic genes. On the other hand, GCs efficiently suppress classical pro-inflammatory macrophage (M1) activation, as evidenced by the inhibition of the pro-inflammatory cytokines TNF, interferon gamma (IFNγ) and IL-1β (64–67) (Figure 2). These cytokines are highly upregulated in many inflammatory disorders, and their crucial role in the pathogenesis of IBD is well-established (68, 69). GCs potently inhibit the differentiation of DCs and their capacity to stimulate T cells (70, 71).

The resulting immune reaction in pathophysiological conditions depends on the balance between effector cells promoting inflammation and its modulation by regulatory mechanisms (72). In this context, the discussed anti-inflammatory and immunosuppressive properties of GCs are necessary to restore homeostasis following successful elimination of the injurious agent, ultimately leading to the resolution of inflammation and tissue repair after tissue damage caused by excessive inflammation (12).

Another main biological function of adrenal GCs includes the control of energy metabolism and glucose homeostasis. GCs promote gluconeogenesis in the liver and decrease glucose uptake by antagonizing the response to insulin. Whereas, physiological levels of GCs are required for proper metabolic control, excessive GC action has been linked to a variety of metabolic diseases, such as type II diabetes and obesity (73, 74).

## Extra-adrenal GC Synthesis

### Overview of Extra-Adrenal GC Synthesis

The substantial capacity of the adrenal glands to produce enormous amounts of GCs and to release them into the systemic circulation in response to stress hampered the discovery of other GC-producing organs. In fact, strong systemic immune cell activation upon removal of the adrenal glands in mice results in rapid death due to shock (75). Therefore, for long time GC synthesis and secretion was thought to be exclusively confined to the adrenal glands. However, increasing evidence has shown that other extra-adrenal organs are also capable of producing GCs [reviewed in (76)]. Evidence for local GC synthesis comprises the detection of steroidogenic enzymes and high levels of local GCs in different tissues, even upon adrenalectomy. Moreover, the physiological relevance of local GC synthesis has been shown by the major impact of the inhibition of local GCs synthesis even in adrenal-intact scenarios (76–79). Thus, whereas systemic adrenal-derived GCs coordinate multiple organ functions and whole body metabolism, locally synthesized GCs play a highly specific role in regulating local homeostasis, cell development and immune cell activation (31, 80).

In the past two decades the thymus (81–83), the skin (84, 85), the brain (78), the vasculature (86), the lung (79), and the intestine (77, 87–90) have been shown to produce substantial amounts of GCs, and thereby regulate local immunological responses.

Pioneering work by the group of Ashwell in the thymus provided the first proof for extra-adrenal GC production and opened an exciting field of research for the identification of other GC-producing organs (83). They showed that bioactive GCs are *de novo* synthesized by thymic epithelial cells (TECs), and that they play an important role in antigen-specific thymocyte development by opposing cell death induction from too strong TCR signaling during negative selection, thereby allowing positively selected T cells to survive. This is supported by the finding that inhibition of thymic corticosterone production increased TCR activation-induced cell death and enhanced negative selection of thymocytes (13, 58, 83). Of note, thymocytes (91) and mature T cells (92, 93) were also reported to synthesize GCs, yet it is presently unclear whether this reflects *de novo* synthesis or conversion of serum-derived inactive derivatives.

Interestingly, the skin locally produces CRH, ACTH and expresses the steroidogenic enzymes. Therefore, the skin is considered to have its own local HPA axis. *De novo* synthesis of GCs in the skin is thought to play an important role in local homeostasis as indicated by the deficiency of the steroidogenic enzymes in skin biopsies from patients with inflammatory skin diseases (94). Other organs that express the GC-synthesizing machinery and therefore are capable to *de novo* synthesize bioactive GCs from cholesterol include the brain, the vasculature and the intestine (95). Interestingly, although the lung expresses all the steroidogenic enzymes required for *de novo* synthesis, analysis of lung GC synthesis revealed that the predominant pathway by which corticosterone is produced is by reactivation from inactive serum-derived dehydrocorticosterone via 11β-HSD1 enzyme (79).

### Differential Modes of Synthesis of Extra-Adrenal GCs

Most of extra-adrenal GC-synthesizing organs express both the enzymes required for *de novo* GC synthesis as well as the reactivating enzymes from inactive metabolites. However, interestingly different extra-adrenal organs synthesize bioactive GCs via different mechanisms, possibly reflecting local environmental needs. For example, TECs have been shown to have the mRNA, protein, and activities of enzymes required for *de novo* GC synthesis, including StAR, CYP11A1, 3β-HSD, CYP17, and CYP11B1. Furthermore, fetal thymic organ culture demonstrated the conversion of a cholesterol analog to pregnenolone and 11-deoxycorticosterone. Similar to adrenal GCs, TEC-derived GC synthesis was stimulated by ACTH (76, 83). In contrast, ACTH inhibited GC synthesis in thymocytes by downregulation of *Cyp11b1* mRNA expression. This opposite effect of ACTH in thymocytes is not yet fully understood but could possibly represent a function to limit damage to the gland by down-regulating GC synthesis during a strong activation of the HPA axis (91).

Like the thymus, the skin mainly synthesizes GCs *de novo* under the control of the local HPA axis, and the synthesis is regulated by several factors including ACTH, CRH and IL-1β (85). Noteworthy, the skin neuroendocrine system is able to crosstalk with the systemic HPA axis and thus with the adrenal GC synthesis. Interestingly, although the skin also expresses the reactivating enzyme 11β-HSD1, GC reactivation by keratinocytes seems to play a minor role in immune cell activation and contact hypersensitivity compared to the essential role of the *de novo* synthesized GCs. The reason for this could be due to its large dependence on the availability of the GC metabolite from the circulation (94).

As mentioned before, the lung largely depends on the reactivation pathway for generating bioactive GCs. Our group reported that upon immune cell activation by lipopolysaccharide (LPS) or anti-CD3 antibody increased production of corticosterone in *ex vivo* lung cultures was observed (79). Interestingly, only *Cyp11a1* has been shown to be upregulated whereas other steroidogenic enzymes expression remained unchanged. Strikingly, whereas *Hsd11b1* gene was strongly upregulated, *Cyp11b1* was barely detectable indicating that reactivation of serum-derived inactive metabolite (dehydrocorticosterone) is a more prominent pathway of local GC synthesis in the lung. In line with this, adrenalectomized mice failed to produce local GCs in the lung upon immune cell activation. This finding further supports the dependence of lung GC synthesis on adrenal GCs (79).

Our group also described and characterized the *de novo* synthesis of intestinal GCs for the first time. In the intestinal mucosa, GC-synthesizing enzymes were detected at low levels, however, they were strongly upregulated in response to immunological stress resulting in the detection of corticosterone in the supernatant of *ex vivo* cultured intestinal tissue (77). Recently, we also demonstrated a relevance for the GC reactivation impairment in the pathogenesis of IBD (96). In this review, we will discuss the synthesis of GCs in the intestine in more detail.

Taken together, it seems that various extra-adrenal organs synthesize GCs differently in order to cope with local immunological stress and to regulate local immune homeostasis.

## Intestinal Epithelial Structure and Homeostasis

The intestinal epithelium represents the largest mucosal surface in the human body covering an area of almost 200 m^2^ (21). This surface represents the physical barrier that separates the epithelium not only from potential pathogens and food antigens but also from harmless commensal bacteria termed microbiota (97, 98). The gut is anatomically divided into the small intestine and the colon. The small intestine can be subdivided into the duodenum, the jejunum, and the ileum. The architecture of the intestine is organized into crypts of Lieberkühn and epithelial protrusions, called villi, in the small intestine, whereas the colon consists mainly of crypts and has no villi, but a flat surface instead (99, 100). The main function of the epithelium is water and nutrient absorption and the maintenance of effective barrier function in order to maintain tissue homeostasis (101, 102). The intestinal epithelium promotes these functions by a single layer of intestinal epithelial cells (IECs) organized along the crypt-villus axis (99, 103). IECs are constantly regenerated from intestinal stem cells (ISCs) at the bottom of the crypt columnar cells. The intestinal epithelium has a higher self-renewal rate than any other mammalian tissue, with a fast turnover of <5 days (104–106). ISCs give rise to transient amplifying daughter cells that migrate upward while differentiating into one of the specialized epithelial lineages (100). This fast proliferation of IECs is eventually balanced by cell death resulting from loss of attachment at the tip of the villus followed by subsequent shedding of apoptotic cells into the lumen, a process known as anoikis (106). The differentiated epithelial cell types include absorptive enterocytes, secretory cells (Paneth cells, goblet cells, enteroendocrine cells, and tuft cells), and the M cells of Peyer's patches (105). Paneth cells escape the upward migration and migrate downward instead to constitute the niche for ISCs (107). These cells secrete anti-microbial peptides to prevent bacterial infection (103), whereas tuft cells act as sensors for luminal contents (108). Additionally, enteroendocrine cells secrete various hormones to coordinate digestion and metabolism (108).

Contributing to the effective physical and biochemical barrier function is the mucus secreted by goblet cells, anti-microbial proteins that eliminate bacteria penetrating the mucous and IgA secreted by lamina propria plasma cells, in addition to the tight junctions (TJ) proteins. These TJ are junctional complexes that connect epithelial cells to each other and thereby forming tight intracellular seals (109, 110). The intestinal mucosa also produces high levels of the immunosuppressive cytokines transforming growth factor beta (TGFβ) and IL-10 to maintain local homeostasis. In fact, TGFβ- and IL-10-deficient mice develop spontaneous inflammation (111, 112).

IECs separate the intestinal lumen containing 10^14^ gut microbiota cells from the underlying lamina propria and the rest of the body (113, 114). In addition to the microbiota, the gut epithelium hosts the largest number of immune cells in the body (115). These immune cells include the so-called intraepithelial lymphocytes (IELs) (116), resident macrophages, DCs, plasma cells, lamina propria lymphocytes (LPLs), and neutrophils (115, 117, 118). This direct contact of immune cells with the microbiota, that has great potential to provoke immune cell stimulation, requires fine-tuning to find the appropriate balance between protective immune responses and tolerance toward the microbiota. Disruption of the intestinal epithelial barrier leads to permeability defects, and subsequent interaction between luminal microorganisms and cells of the immune system (Figure 2). The barrier breakdown exacerbates inflammation leading to severe tissue damage, as in the case of IBD (98).

IBD comprises a group of intestinal inflammatory disorders, namely ulcerative colitis (UC) and Crohn's disease (CD). Although the etiology is currently not fully understood, it has been associated with a complex interaction between the host genetics, environmental or microbial factors and the immune system (119–121). These interactions result in chronic relapsing inflammation of the intestine as a consequence of inappropriate immune cell activation (117). UC causes inflammation of the mucosa of the colon and rectum, whereas CD causes inflammation of the full thickness of the bowel wall and may involve any part of the digestive tract from the mouth to the anus (122).

Chronic inflammation has emerged as one of the hallmarks of cancer. Many cancers arise following prolonged inflammation or display inflammatory characteristics throughout progression (123, 124). For example, the relative risk of colorectal cancer in patients with IBD has been estimated to increase by up to 20-fold (125, 126). Notably, the risk correlates directly with the duration and extent of inflammation (127, 128).

Increasing lines of evidence have shown that the synthesis of GCs by IECs plays an important role in the regulation of intestinal immune homeostasis under pathophysiological conditions (21, 77, 129, 130). Supporting this notion, defective local intestinal GC synthesis or metabolism has been shown to be involved in the pathogenesis of intestinal inflammation (90, 96, 131, 132).

## Extra-adrenal Glucocorticoids in the Intestine

First evidence for the steroidogenic potential of the gut was suggested in 1995 following the detection of *Cyp11a1* and *Hsd3b1* mRNA in the gut of mouse embryos by *in situ* hybridization (133). Further evidence originated from our own work while studying IEL apoptosis. It was observed that IELs rapidly undergo apoptosis when cultured *ex vivo*, an effect that was accelerated following GC treatment in mice. Interestingly, while adrenalectomy significantly reduced IEL *ex vivo* apoptosis, a stronger effect was observed upon *in vivo* administration of the GR inhibitor RU-486. This observation prompted us to speculate that another source of GCs, likely in the intestinal mucosa, primed the IELs already *in vivo* to undergo *ex vivo* cell death (56).

Subsequent studies characterized the *de novo* GC synthesis in the murine intestinal mucosa in response to immunological stress following anti-CD3 injection or viral-activated T cells (77). It was shown that the intestinal mucosa constitutively expressed many of the steroidogenic enzymes required for the *de novo* synthesis of corticosterone from cholesterol and for the reactivation of corticosterone from dehydrocorticosterone. Moreover, expression of the steroidogenic enzymes including *Cyp11a1, Cyp11b1*, and *Hsd11b1* was strongly induced upon immunological stress. The source of the aforementioned three enzymes and therefore intestinal GCs was shown to be the crypt region of the IECs (77). This was demonstrated by a further study that linked the expression of *Cyp11a1* and *Cyp11b1* to the cell cycle, thus restricting the production of GCs to the proliferating cells of the intestinal crypts (134).

The basal expression of steroidogenic enzymes might suggest that GC production, though at very low levels, is possibly fulfilling an important function in the regulation of local immune homeostasis and epithelial barrier integrity (75). In line with this, *in vitro* data revealed the importance of GCs in the maturation and differentiation of the IECs (135). Additionally, GCs have been shown to play a role in the expression of TJ proteins and the maintenance of the intestinal epithelial barrier integrity, in particular antagonizing the TJ-destructing effect of TNF during inflammation (109) (Figure 2).

Cima et al. used adrenalectomized mice to exclude the contribution of systemic GCs, and measured by radioimmunoassay the corticosterone release into the supernatant of *ex vivo* cultured intestinal tissue from anti-CD3-injected mice (77). The *in situ* corticosterone synthesis was confirmed since metyrapone, a potent inhibitor of 11β-hydroxylase and 11β-HSD1 (136, 137), blocked corticosterone release (77). Similarly, stimulation of the innate immune system with LPS induces GC synthesis in a macrophage-dependent manner, since it also occurred in RAG^−/−^ mice lacking T and B lymphocytes (89). Furthermore, administration of TNF, infection of mice with viruses, or chemically induced intestinal inflammation promote the expression of *Cyp11a1* and *Cyp11b1*, and strongly induces the synthesis of intestinal GCs (95). Although most of the studies of GC synthesis were conducted in mice, subsequent research showed that the human intestinal tissue also expresses the steroidogenic enzymes and is capable of synthesizing GCs (96, 138–140).

### Intestinal GC Triggers and the Role of TNF

TNF is a pro-inflammatory cytokine with a wide range of pleiotropic functions. TNF interacts with two different receptors, designated TNF receptor (TNFR) 1 and TNFR2, which are differentially expressed on cells and tissues, and initiate both distinct and overlapping signal transduction pathways. These diverse signaling cascades lead to a range of cellular responses, which include cell death, inflammation, survival, differentiation, proliferation, and migration (141, 142). In the intestinal epithelium, TNF demonstrates variable and very complex functions in physiological as well as pathological conditions (143). TNF has been shown to drastically promote epithelial cell death (144) and increase the epithelial barrier permeability via a direct effect on the expression and organization of TJ proteins, thereby leading to intestinal inflammation (Figure 2). In fact, TNF is considered as one of the most important effector molecules in the pathogenesis of IBD (145). Moreover, TNF signaling has been shown to drive colonic tumor formation after sustained chronic colitis. Consequently, TNFR deficiency or the treatment of wild type mice with the specific pharmacological inhibitor of TNF, etanercept, markedly reduces colitis-associated colon cancer (146).

Although the main cellular source for TNF is immune cells, fibroblasts and epithelial cells have also been shown to produce TNF (147). Macrophage and T cell activation results in massive release of TNF, which contributes to the damage of the epithelial layer (148). Therefore, TNF-neutralizing antibodies have been efficiently used for the treatment of IBD (142, 149). This is mainly due to inhibition of IEC cell death, but also due to the downregulation of pro-inflammatory processes that might contribute to local tissue damage (101) (Figure 2).

Despite the well-characterized pro-inflammatory properties of TNF, accumulating evidence for anti-inflammatory roles of TNF is increasingly appreciated. For example, Naito et al. demonstrated that the absence or neutralization of TNF in a mouse model of dextran sulfate sodium (DSS)-induced colitis exacerbated intestinal inflammation (150). Further studies revealed that TNF induces intestinal GC synthesis by direct activation of IECs, thus contributing to intestinal immune homeostasis. In this regard, TNF plays an anti-inflammatory role (90) that could be in part through sensitizing activated T cells to undergo apoptosis, thus resulting in accelerated resolution of the inflammation (151). Interestingly, TNF seems to be the master regulator of intestinal GC synthesis irrespective of the trigger (Figure 2). Noti et al. investigated the intestinal GC synthesis following macrophage and T cell activation in TNFR-deficient and wild type mice. They showed that, while immune cell activation resulted in robust induction of intestinal GCs in wild type mice, it was significantly decreased in TNFR-deficient mice (89). Similarly, intestinal GC synthesis was lacking in mice with TNF deficiency or in TNFR-deficient mice treated with the inflammatory agent DSS or the hapten 2,4,6-trinitrobenzenesulphonic acid (TNBS). In marked contrast, oxazolone, a hapten that promotes a Th2 cytokine-mediated intestinal inflammation that does not involve TNF, fails to promote intestinal GC synthesis (90). These observations clearly indicate that inflammation *per se* is not sufficient to promote intestinal steroidogenesis, but rather the type of inflammation appears to be critical. It also points out the dependence of intestinal GC synthesis on TNF (90, 95).

Taking into consideration the mutual antagonistic action of TNF and GCs, this GC-regulatory function of TNF might appear confusing at a first glance. Nevertheless, local intestinal GC synthesis may counterbalance the deleterious effects of TNF in two ways: (1) an increase in barrier resistance by promoting the expression of TJ proteins and (2) by dampening overwhelming immune responses and the associated immune cell activation that are triggered by epithelial barrier disruption. Hence, although TNF is involved in the disruption of the epithelial barrier integrity, it is also involved in restoring intestinal epithelial barrier function by the induction of GC synthesis as a negative feedback loop (Figure 1). Moreover, since TNF is not only produced by immune cells but also by IECs, it is feasible to believe that this regulatory system may even work in an epithelial layer-autonomous manner (75, 89).

Taken together, TNF seems to function as a sensor of intestinal immune responses and a master regulator of intestinal GC synthesis in response to activation of the innate and adaptive immune system. Furthermore, TNF mediates a novel anti-inflammatory function via the induction of intestinal GC synthesis (89) (Figure 2).

### Intestinal GCs Functions

Under steady-state conditions, GCs have been implicated in the maturation and the maintenance of the intestinal epithelial barrier integrity. For instance, results from *in vitro* experiments revealed that synthetic GCs had a protective effect against the TNF-dependent increase of intestinal permeability. Microarray data analysis demonstrated that GCs differentially regulate the expression of enterocyte markers that are involved in the polarization and TJ formation (152).

Given the potent immunoregulatory activities of GCs, extra-adrenal GC synthesis in the intestine is assumed to play an important role in the regulation of local immune homeostasis. Indeed, in the intestinal mucosa GCs are synthesized in response to immunological stress. Local GCs then inhibit the activation of immune cells in a negative feedback leading to the resolution of inflammation and associated tissue damage (77, 89, 90). Following anti-CD3 antibody injection, *in situ* produced GCs exhibited a regulatory activity on intestinal T cells that are in close contact with the GC-producing IECs, i.e., IELs and Peyer's patches lymphocytes (PPLs) (77). Likewise, infection of mice with the lymphocytic choriomeningitis virus (LCMV) results in the activation and expansion of virus-specific intestinal T cells and the subsequent release of GCs. GCs in turn suppress anti-viral immune responses. In fact, inhibition of intestinal GC synthesis accelerated the expansion of antigen-specific cytotoxic T cells, further confirming the immunoregulatory role of locally produced GCs (77, 130).

In another study, experimental colitis induction via DSS or TNBS resulted in epithelial erosion, loss of goblet cells, and strong immune cell infiltration into the intestinal mucosa. Simultaneously, it promoted the upregulation of pro-inflammatory mediators such as TNF, steroidogenic enzymes and the synthesis of intestinal GCs. Notably and in line with the discussed role of TNF in the induction of intestinal GC synthesis, the injection of TNF triggered intestinal GC synthesis and resulted in the amelioration of oxazolone-induced colitis in mice. Interestingly, inhibition of intestinal GC synthesis by metyrapone abrogated the observed anti-inflammatory effect of TNF (89).

More recently, in a mouse model of DSS-induced colitis, mice with IEC-specific deletion of the microsomal P450 reductase enzyme (null mice) exhibited a significant decrease of colonic GC synthesis compared to wild type mice. This was associated with an exacerbated colonic inflammation, as evidenced by the presence of higher levels of pro-inflammatory cytokines, increased weight loss, colon shortening and colonic tissue damage in the null mice. Remarkably, restoration of colonic GC synthesis resulted in amelioration of the colitis (153). This clearly indicates that intestinal GCs are synthesized as a mechanism to counterbalance local inflammation. Supporting this notion, the expression of *CYP11A1* and *CYP11B1* were robustly reduced in the inflamed colon biopsies of patients with IBD compared to healthy controls (138).

Furthermore, intestinal GCs critically regulate the expression of colonic peroxisome proliferator-activated-receptor-gamma (PPARγ). PPARγ is a critical regulator of the inflammatory responses by transrepressing TFs, such as NF-κB and AP-1. Consequently, disruption of PPARγ expression in mouse colonic epithelial cells increases susceptibility to DSS-induced colitis. In line with the anti-inflammatory role of PPARγ, reduced expression was observed in IBD patients. That also correlated with a significant reduction in colonic GC synthesis and the expression of steroidogenic enzymes (140).

We recently demonstrated a significant downregulation of *HSD11B1* gene expression, with a simultaneous upregulation of *HSD11B2*, in colons from pediatric IBD patients compared to healthy controls (96). This opposite transcriptional regulation of 11β-HSD isoenzymes could indicate a possible role of defective local GC reactivation in the pathogenesis of IBD by limiting the local levels of the active immunomodulatory GCs, thus hindering the resolution of inflammation. However, in a murine model of acute colitis we observed the opposite, where we found a significant upregulation of *Hsd11b1* and a downregulation of *Hsd11b2* upon colitis induction (96). Interestingly, these correlations were also reported when comparing inflamed tissue to non-inflamed colonic tissue in IBD patients, suggesting that dysregulation of the 11β-HSD enzyme system could play a role in the pathogenesis of IBD (132, 154). Taken together, in view of the discussed immunoregulatory roles of intestinal GCs, it is conceivable to believe that defective intestinal GC synthesis represents a potential key mechanism in the pathogenesis of IBD.

### Intestinal GC Synthesis Regulation

#### Transcriptional Regulation

Whereas, the regulation of adrenal GC synthesis has been extensively studied and most of the pathways are well-defined, the molecular pathways for the regulation of extra-adrenal GC synthesis await further investigation (21). Mueller et al. investigated the molecular basis of steroidogenesis in the intestine and found substantial differences in the mode of regulation of intestinal GC synthesis as compared to the adrenals. This distinct regulation of intestinal GC synthesis could possibly reflect an adaptation to the local environment (88). For example, in marked contrast to the well-known regulatory role of SF-1 in adrenal GC synthesis [reviewed in (26)], SF-1 expression was found to be absent in the intestine. Interestingly, SF-1 activity was replaced by its close homolog, the NR liver receptor homolog-1 (LRH-1, NR5A2) (87, 88).

LRH-1 is expressed in tissues derived from endoderm, including intestine, liver, exocrine pancreas, and the ovary (155). Moreover, LRH-1 is expressed in macrophages (156) and T cells (157). LRH-1 plays vital roles in early embryonic development as evidenced by the embryonically lethal phenotype of the LRH-1-null mice (158). Other functions of LRH-1 comprise cholesterol and bile acid homeostasis, glucose metabolism and steroidogenesis in adulthood (159, 160). In the intestinal epithelium, LRH-1 contributes to crypt cell proliferation and epithelial cell renewal through the induction of cell cycle genes, namely cyclin D1 and cyclin E1 (161). Therefore, LRH-1 has been suggested as an oncogene and implicated in the development of colon cancer (162).

LRH-1 is constitutively active, though its function is regulated by several mechanisms. These include ligand binding, interactions with co-activators and co-repressors, as well as posttranslational modifications, such as phosphorylation and SUMOylation (160, 163, 164). Although LRH-1 is considered as an orphan NR since no endogenous ligands are identified yet, phospholipids such as dilauroyl phosphatidylcholine (DLPC) have been shown to activate LRH-1. Thus, it is very likely that endogenous ligands exist (165, 166). Among the most studied co-repressors of LRH-1 is the NR small heterodimer partner (SHP) (167), which is also a transcriptional target of LRH-1 (168). Structural studies have shown that SHP preferentially inhibits LRH-1 over other NRs, including the LRH-1 close homolog SF-1 (169, 170).

#### Differences in Regulation of Intestinal vs. Adrenal GC Synthesis

The differential regulation of intestinal vs. adrenal GC synthesis, i.e., LRH-1 vs. SF-1, is likely reflecting different needs for the systemic vs. intestinal GC synthesis (21). In this regard, another major difference is the differential response of adrenal and intestinal epithelial cells to cAMP and phorbol myristate acetate (PMA). In the adrenals, it is well-established that the activation of ACTH receptors leads to the activation of adenylate cyclase and the formation of cAMP. In turn, cAMP activates protein kinase A leading to the induction of steroidogenic enzyme expression. Surprisingly, cAMP mediated the opposite effect in intestinal epithelial cells by causing a profound inhibition of both basal and LRH-1-driven steroidogenesis. Remarkably, a reciprocal effect was shown upon treatment with PMA that activates protein kinase C. PMA has been shown to substantially promote both basal and LRH-1-induced steroidogenic enzymes expression and GC synthesis in intestinal epithelial cells (88). As PMA is a potent activator of the MAPK pathway, it is likely that PMA affects LRH-1 activity by inducing its phosphorylation (21, 171).

#### LRH-1 Function in Intestinal Homeostasis

In the murine intestinal epithelial cell line mICcl2, that displays a crypt cell-like phenotype, overexpression of LRH-1 induced the expression of *Cyp11a1* and *Cyp11b1* in a dose-dependent manner. This was accompanied by robust induction of GC synthesis (87). Since LRH-1 is critical for embryonic development, Mueller et al. used LRH-1 haplodeficient mice to investigate the role of LRH-1 in the regulation of intestinal GCs *in vivo*. They showed that although anti-CD3 injection strongly induced the expression of *Cyp11a1* and *Cyp11b1*, and the synthesis of intestinal GCs in wild type mice, it was blunted in LRH-1 haplodeficient mice. These findings confirm the critical role of LRH-1 in the regulation of intestinal GC synthesis (87).

**Figure 3:**
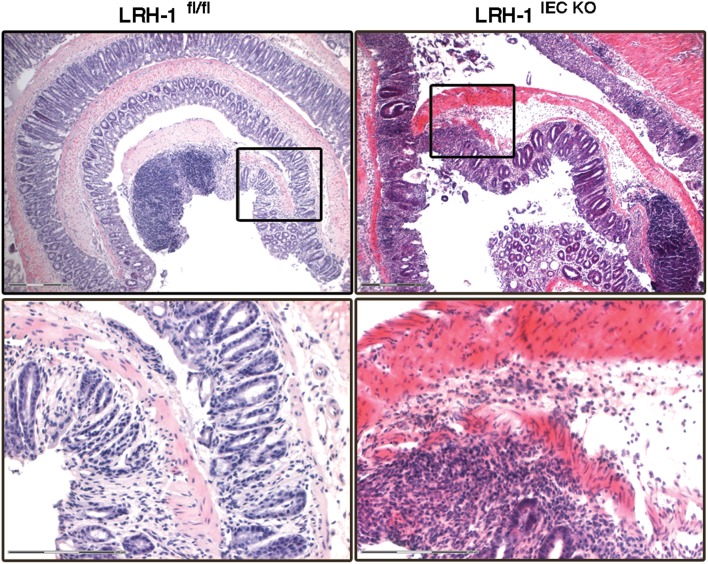
LRH-1 is critical for intestinal immune homeostasis. Colitis was induced in female 8–9 weeks-old wild type (LRH-1^fl/fl^) mice and intestine epithelial cell-specific knockout mice (LRH-1^IEC KO^) by administration of 2.2% (w/v) DSS in the drinking water for 5 days followed by normal drinking water for 2 days. Representative H&E staining of Swiss-rolled colon sections of mice treated with DSS at day 7 showing the exacerbated colitis in LRH-1^IEC KO^ mice compared to LRH-1^fl/fl^ mice. Scale bars: 300 μm overview, 150 μm inlay.

In humans, LRH-1 transcriptionally regulates the expression of the steroidogenic enzymes CYP11A1, CYP17, HSD3B2, and CYP11B1 as well as StAR (172). The importance of LRH-1 in the regulation of intestinal GC synthesis and intestinal immune homeostasis has been demonstrated by the fact that LRH-1 haplodeficient mice and mice with intestine-specific deletion of LRH-1 exhibited strongly reduced GC synthesis, and consequently suffered from exacerbated colitis (87, 96, 138) (Figure 3). Furthermore, colon biopsies from patients with IBD show reduced expression of LRH-1 and steroidogenic enzymes. That was inversely correlated with the expression of pro-inflammatory cytokines (138). Additionally, it has been shown that cortisol production and the expression of LRH-1 and 3β-HSD1 were significantly decreased in colonic epithelial cells from patients with UC (140). Recently, we demonstrated a strong correlation between the expression of LRH-1 and steroidogenic enzymes in pediatric IBD patients (96). Importantly, we monitored a significantly reduced expression of *HSD11B1* in colons from IBD patients compared to healthy controls suggesting that defective reactivation of GCs could represent an underlying mechanism in intestinal inflammation. Additionally, in a murine model of colitis we confirmed that colitis-induced expression of the steroidogenic enzymes *Cyp11a1, Cyp11b1*, and *Cyp21* is LRH-1-dependent since their induction was significantly reduced in LRH-1 intestine-specific knockout mice (96). These data suggest that the presence of LRH-1 protects the intestinal epithelium against inflammation and underscores a possible role for defective local GC synthesis in the etiology of IBD.

Interestingly, SHP inhibits LRH-1-induced *Cyp11a1* and *Cyp11b1* expression and GC synthesis in mICcl2 cells (88). This indicates a potential role of SHP in the regulation of intestinal immune homeostasis by regulating LRH-1-induced GC synthesis. Recently, Huang et al. investigated the role of the NRs SHP and LRH-1 in the regulation of intestinal GC synthesis and its relevance in intestinal immune homeostasis in the context of viral infection (130). They showed that systemic deficiency of SHP results in increased intestinal GC synthesis during viral infection that suppressed the expansion and activation of virus-specific T cells. In contrast, intestine-specific deletion of LRH-1 strongly reduced intestinal GC synthesis and accelerated the expansion of cytotoxic T cells upon viral infection (130). Noteworthy, Bayrer et al. recently showed that intestinal organoids lacking LRH-1 exhibit reduced expression of the LRH-1 target genes *Shp, Cyp11a1*, and *Cyp11b1*, as well as increased crypt cell death and epithelial permeability (173). They also showed that overexpression of LRH-1 mitigated inflammation-induced damage of murine and human intestinal organoids, including those from IBD patients, and decreased the disease severity in a T cell transfer model of colitis (173).

Of note, the expression of steroidogenic enzymes is linked to the cell cycle, thus implicating a restriction of the intestinal GC synthesis to the proliferating cells at the bottom of the crypts (134, 152). Similar to steroidogenic enzymes, LRH-1 expression is confined to the proliferating cells of the crypts, suggesting a cell cycle-dependent regulation of intestinal GC synthesis (87, 134, 161).

**Figure 4:**
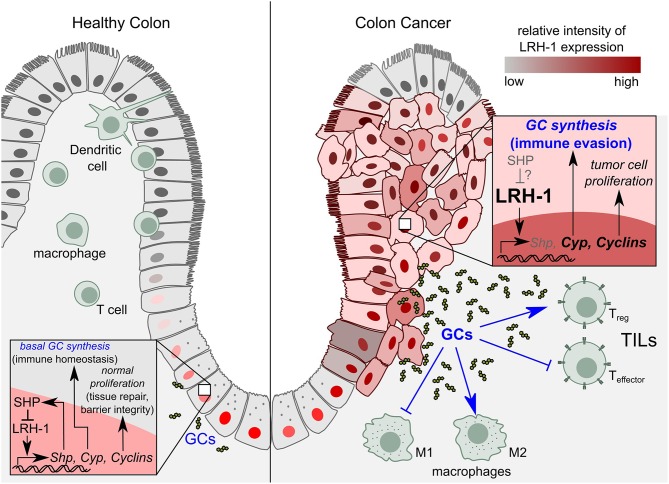
Role of LRH-1 in healthy colon vs. colon cancer. Left panel: In healthy colon, LRH-1 is expressed in the nucleus of cells at the bottom of the intestinal crypts, where it regulates intestinal immune homeostasis by the regulation of cell proliferation through cyclins on the one hand, and the synthesis of immunoregulatory glucocorticoids (GCs) on the other hand. Right panel: In colon cancer, LRH-1 exhibits a nuclear as well as cytoplasmic expression pattern. LRH-1 induces colon tumor cell proliferation by upregulating expression of cyclins. LRH-1 is proposed to play a role in tumor immune evasion by the synthesis of immunosuppressive GCs that leads to the inhibition of anti-tumor immune responses. While in healthy tissue SHP imposes a negative feedback loop to LRH-1 signaling, the role of SHP in the molecular events during colon cancer development remains to be elucidated. SHP, Small heterodimer partner; TILs, tumor infiltrating lymphocytes; T_reg_, regulatory T cells; T _effector_, effector T cells; M1, pro-inflammatory macrophages (anti-tumor); M2, anti-inflammatory (tumor promoting) macrophages.

LRH-1 seems to contribute to intestinal epithelium homeostasis via two mechanisms: (1) by stimulating the synthesis of anti-inflammatory GCs and thereby resolution of inflammation and associated tissue damage, (2) by enhancing crypt cell proliferation and hence the regeneration of the damaged epithelium (Figure 4).

Interestingly, LPS-induced GC synthesis seems not to be regulated by LRH-1, since it was not affected by LRH-1 deficiency. Surprisingly, LRH-1 haplodeficient mice expressed even higher levels of *Cyp11b1* and showed a tendency toward increased GC synthesis in response to LPS exposure compared to wild type mice (89). This clearly indicates that other signals and TFs are regulating GC synthesis in response to innate immune system stimulation. Furthermore, TNF has been shown to suppress LRH-1 and thereby reduce local GC synthesis in sustained chronic colitis (174).

Of interest is the finding that under basal conditions the microbiota also contribute to the regulation of intestinal GC synthesis. Furthermore, intestinal GC synthesis has been shown to regulate systemic metabolism, indicating a so far unrecognized role for intestinal GC synthesis in not only regulating local but also systemic homeostasis (114).

In summary, despite the well-established roles of TNF and LRH-1 in the regulation of intestinal GC synthesis, their interaction in this process is still unclear. It could be possible that multiple pathways and interaction partners are involved in LRH-1-regulated intestinal GC synthesis. Moreover, we cannot exclude that TNF and LRH-1 are acting via independent mechanisms to stimulate intestinal GC synthesis. Nonetheless, our understanding of these interactions is far from being established and other regulatory mechanisms for intestinal GC synthesis are yet to be defined. It would also be relevant to investigate the possible crosstalk between local intestinal GCs and systemic GCs, and how this is regulated.

## LRH-1 in Intestinal Tumors

In the intestinal epithelium, LRH-1 regulates not only steroidogenesis (87, 89), but also crypt cell proliferation (161). Thus, LRH-1 has been shown to contribute to intestinal tumor formation (162) (Figure 4). LRH-1 induces cell proliferation through the concomitant induction of the cell cycle-regulating gene products cyclin D1 and E1, and c-Myc, which is further potentiated by its interaction with β-catenin. Whereas, β-catenin co-activates LRH-1 after direct binding of LRH-1 to the cyclin E1 promoter, LRH-1 acts as a co-activator for β-catenin/TCF4 (T cell factor 4) on the cyclin D1 promoter (161, 162). Due to its role in proliferation and the maintenance of pluripotency, LRH-1 has emerged as an oncogene implicated in the development of a variety of cancers, including pancreatic (175), prostate (176), breast (177, 178), gastric (179), and colorectal cancer (CRC) (162, 180). LRH-1 exhibited an increased expression pattern in high-grade prostate cancer, and has been reported to promote prostate cancer growth by inducing intra-tumoral steroidogenesis (176). LRH-1 also contributed to metastasis development in pancreatic cancer (175).

LRH-1 has been shown to drive colon cancer cell growth by repressing the expression of the cell cycle inhibitor p21 in a p53-dependent manner (180). Consistent with the role of LRH-1 in CRC development, it has been shown that LRH-1 heterozygous mice developed significantly less tumors compared to wild type in two independent models of CRC, the azoxymethane-induced and APC^min/+^ mice model (162). Unlike the nuclear expression of LRH-1 at the bottom of the normal colonic crypts, immunostaining of neoplastic colon from patients with high-degree dysplasia showed significantly higher cytoplasmic levels. Additionally, in neoplastic lesions, staining of LRH-1 was no longer limited to the cells lining the crypts but also present in the surface epithelial cells (Figure 4). These alterations in LRH-1 expression and subcellular localization further indicate the important role of LRH-1 in CRC development (162). Moreover, if and how the LRH-1-induced SHP, which in healthy colon tissue counterbalances LRH-1 function, contributes to the molecular events during colon cancer development, remains unknown (Figure 4).

In contrast to the known role of LRH-1 in intestinal tumorigenesis, LRH-1 expression has been shown to be significantly downregulated in murine adenoma tissue compared to adjacent normal mucosa. The expression of LRH-1 gene was reduced in tumors that express elevated levels of the pro-inflammatory cytokine TNF. Reciprocally, decreased LRH-1 expression in heterozygous mice attenuates TNF expression (162). However, the relevance of this inverse correlation is so far unknown and again points out the complex interaction between TNF-induced signaling pathways and LRH-1.

Recently, a large CRC patient cohort revealed that immunohistochemical detection of LRH-1 expression was drastically enhanced in colon cancer tissue compared to adjacent non-cancerous tissue from the same patient, and this correlated with a more advanced disease stage. In fact, patients with positive LRH-1 expression displayed significantly lower overall survival rate. Consequently, the authors proposed LRH-1 as a possible prognostic marker and a novel therapeutic target in CRC (181). These observations were confirmed in another recent study that revealed marked overexpression of LRH-1 in CRC tissue compared to paired non-cancerous tissue (182). Taken together, LRH-1 represents a novel and promising therapeutic target for the treatment of cancer.

## Intestinal GC Synthesis as a Tumor Immune Escape Mechanism

The notion that the immune system can recognize and destroy transformed cells is known as cancer immune surveillance. However, since the role of the immune system in controlling cancer growth and recurrence remains highly controversial, this term has been replaced by “cancer immunoediting” to describe the dual roles of the immune system in promoting host defense and facilitating tumor growth and immune escape (183, 184). Several mechanisms by which cancer cells evade the immune system have been described. These include: (1) immune suppression at the tumor microenvironment mediated by Tregs or other types of suppressive cells (the major mechanism of tumor immune escape), (2) induction of apoptosis in tumor-specific cytotoxic T lymphocytes (CTLs) by the expression of pro-apoptotic ligands e.g., Fas ligand and TRAIL, (3) defective antigen presentation, (4) release of immunosuppressive cytokines such as IL-10 and TGFβ, and (5) inducing tolerance and immune deviation by mechanisms including, among others, shifting the balance of Th1 immune responses to Th2, and expression of immune inhibitory molecules such as PD-1 (programmed death-1) and CTLA-4 (CTL antigen-4) (95, 185).

Colorectal tumors are highly immunogenic. Therefore, anti-tumor immune responses may significantly limit tumor growth. In fact, a strong correlation between anti-tumor immune responses and CRC patient survival has been demonstrated (186–188). On the other hand, immune escape mechanisms have been recognized as one of the hallmarks of cancer (123, 189). Pagés et al. studied the correlation between pathological signs of early metastatic invasion and the local immune response within the tumor in a cohort of 959 resected colorectal tumors using flow cytometry, gene expression profiling and *in situ* immunohistochemistry (186). In this study, the authors reported up to 15 years clinical follow-up of the patients for the presence or absence of early signs of metastasis. Remarkably, they showed that tumors without such signs had increased infiltrates of CD8+ T cell numbers and increased gene expression for CD8, T-box transcription factor 21, interferon regulatory factor 1, IFN-γ, granulysin, and granzyme B, that correlated with increased survival. Likewise, the presence of high levels of infiltrating memory T cells, as measured by immunohistochemistry, correlated with increased survival (186). The same group confirmed these results in two other independent cohorts of CRC patients (187). Furthermore, in 566 CRC patients a significant positive correlation between markers of innate immune system and early activated T cells has been linked to protection from relapse. Additionally increased densities of CTLs and effector memory T cells within the primary tumor significantly protected CRC patients from tumor recurrence (188). In another study, CRC patients with high expression of Th17 markers had a poor prognosis, whereas patients with high expression of the Th1 markers had prolonged disease-free survival (190). These data provide compelling evidence for the role of the immune system in limiting CRC development and clearly suggest that immune evasion could represent an important mechanism by which colorectal tumor cells prevent their destruction by the immune system.

Supporting this hypothesis, Sidler et al. described the first evidence for a novel LRH-1-dependent GC synthesis in CRC cell lines as well as primary tumors, that exerted inhibitory effects on activated T cells (139). They showed that colon cancer cell lines express the enzymes required for *de novo* synthesis of bioactive GCs, including CYP11A1, CYP11B1, and CYP17. Consequently, cortisol production as measured by thin layer chromatography, radioimmunoassay, and bioassay was detected in culture supernatants (139).

The expression of steroidogenic enzymes in CRC cells is dependent on endogenous LRH-1, as evidenced by the diminished expression of these enzymes upon LRH-1 downregulation. Similar to intestinal GC synthesis, tumor-cell derived GC synthesis was also regulated by LRH-1 since overexpression of LRH-1 boosted cortisol production in a dose-dependent manner, whereas it was significantly inhibited following LRH-1 knockdown. Primary tumors from CRC patients also expressed high levels of LRH-1, CYP11A1, CYP11B1, and StAR, and readily synthesized cortisol following *ex vivo* culture. Interestingly, unlike the basal inducible GC production in the normal intestine, LRH-1-mediated GC synthesis in colonic tumors is constitutive since it was not further enhanced by PMA (139). This observation suggests that LRH-1 is constitutively active, or the presence of LRH-1 activators in the tumor microenvironment. Of interest, enhanced EGF signaling as demonstrated by EGFR overexpression has been shown in 60–80% of CRC patients, that was associated with poor prognosis (191). Since EGF has been shown to exert a mitogenic signal by the MAPK pathway (192), it is tempting to speculate that EGF-induced signaling pathways activate LRH-1 in CRC tumors via a MAPK-induced phosphorylation. However, this hypothesis needs to be further investigated.

Noteworthy, tumor-derived GCs suppressed T cell activation, as shown by the substantial inhibition of CD69 expression (an early activation marker of T cells) in activated CD4^+^ and CD8^+^ murine splenic T cells. This inhibitory effect was GC-specific since it was reversed by blocking the GR (139). Hence, besides its role in inducing tumor cell proliferation, LRH-1 could contribute to CRC tumor development via the synthesis of immunosuppressive GCs (Figure 4). Taken together, LRH-1-mediated synthesis of immunoregulatory GCs in CRC could represent a novel immune escape mechanism by inhibiting T cell-mediated anti-tumor immune responses and thereby favoring the tumor growth.

## Therapeutic Potential and Future Perspective of Intestinal GC Synthesis

### Targeting GCs in Intestinal Inflammation

Thus far, the importance of locally synthesized GCs has been reflected by the impairment of cortisol production as well as decreased LRH-1 expression in colonic epithelial cells from UC patients (138, 140). Despite the advances in introducing novel therapies for the treatment of IBD, GCs remain the first-line treatment for inducing rapid remission in moderate to severe IBD with high efficacy. Nevertheless, emergence of resistance and the side-effects of systemic GCs represent a major therapeutic challenge (131, 193). Along these lines, restoring local GC synthesis in the intestine could represent an attractive approach to ameliorate the symptoms of IBD and to avoid the systemic GC side-effects. This could be achieved by enhancing LRH-1 activity in the intestine since LRH-1 controls both local GC synthesis and epithelial regeneration (87, 89, 90, 138). In fact, a recent study underlined the therapeutic potential of targeting LRH-1 by showing that restoration of LRH-1 reestablished epithelial integrity in mouse and human organoids treated with TNF or 5-fluorouracil, a chemotherapeutic agent with intestinal toxicity. Moreover, overexpression of LRH-1 protected mice from T cell-induced colitis (173). As mentioned earlier, structure-based studies identified DLPC as a potential ligand that was able to enhance LRH-1 transcriptional activity (166). Interestingly, DLPC has been shown to exert anti-diabetic effects by activating LRH-1 in the liver when used in a therapeutic setting (194, 195). Thus, it is tempting to speculate that administration of LRH-1 ligands could also ameliorate intestinal inflammation. However, this attractive idea remains to be tested.

### Targeting GCs in Colorectal Cancer

In CRC, LRH-1 regulates proliferation as well as GC synthesis that could possibly represents an immune escape mechanism (139) (Figure 4). In line with this, LRH-1 has also been described to promote prostate cancer growth by inducing intra-tumoral steroidogenesis (176).

Consistent with the critical role of LRH-1 in tumor development, LRH-1 is overexpressed in many tumors, as discussed above. For instance, a remarkable upregulation of LRH-1 was reported in CRC tissue compared to paired non-cancerous tissue from two independent CRC patient cohorts (181, 182). Hence, suppression of LRH-1 activity in tumors is postulated to exert anti-proliferative effect that could potentially lead to tumor regression. Supporting this notion, LRH-1 knockdown resulted in impaired *in vitro* proliferation of pancreatic and CRC cell lines (175, 196). Recently, Qu et al. showed that targeting LRH-1 via microRNA inhibited *in vitro* proliferation and invasion of CRC cell lines (182). These data provide compelling evidence for the therapeutic potential of targeting LRH-1 in cancer. Advances in structure-based studies identified small molecule inhibitors of LRH-1 including 3d2 (197) and SR1848 (198). The inhibitory effect of 3d2 and SR1848 on LRH-1 was confirmed *in vitro* and *in vivo* and reported to induce anti-proliferative effects on a variety of cancer cell lines (157, 197, 198).

In conclusion, inhibition of LRH-1 activity in colon tumors with high LRH-1 expression represents an interesting therapeutic approach to be followed upon, aiming at inhibition of both LRH-1-induced proliferation as well as GC synthesis. This is of particular interest since in CRC a strong correlation between the degree of immune cell infiltrates and patient survival has been demonstrated (186, 187). Of note, *ex vivo* culture of primary colonic tumors from patients showed increased GC synthesis compared to adjacent non-tumor tissue (139). These observations further underscore that immune evasion, e.g., via the synthesis of immunoregulatory GCs, might be an important mechanism by which intestinal tumors shape the tumor microenvironment resulting on one hand in tumor support by stromal cells, on the other hand in the escape of CRC from the destruction by the immune system.

## Author Contributions

AA and TB designed and discussed the manuscript. AA wrote the manuscript and drafted the figures. CS finalized the figures and revised the manuscript. TB finalized the manuscript.

### Conflict of Interest Statement

The authors declare that the research was conducted in the absence of any commercial or financial relationships that could be construed as a potential conflict of interest.

## Abbreviations

ACTH: Adrenocorticotropic hormone
AF-1: activation function 1
AP-1: activator protein 1
APC: Adenomatous polyposis coli
cAMP: cyclic adenosine monophosphate
CD: Crohn's disease
CREB: cAMP response element binding protein
CRH: corticotropin-releasing hormone
CTL: cytotoxic T lymphocyte
CTLA-4: CTL-antigen 4
CYP: cytochrome P450
DBD: DNA-binding domain
DC: dendritic cell
DLPC: dilauroyl phosphatidylcholine
DSS: dextran sulfate sodium
GC: Glucocorticoids
GILZ: glucocorticoid-induced leucine zipper
GR: glucocorticoid receptor
GRE: GC response element
HPA: Hypothalamus-pituitary-adrenal (gland)
HSD: hydroxysteroid dehydrogenase
IBD: Inflammatory Bowel Disease
IEC: intestinal epithelial cell
IEL: intraepithelial lymphocyte
IFN-γ: interferon gamma
ISC: intestinal stem cell
LCMV: lymphocytic choriomeningitis virus
LBD: ligand-binding domain
LPL: lamina propria lymphocyte
LRH-1: liver receptor homolog-1
MAPK: mitogen activated protein kinase
MC: mineralocorticoid
NF-κB: nuclear factor “kappa-light-chain-enhancer” of activated B cells
NR: nuclear receptor
NR5A1: nuclear receptor subfamily 5 group A member 1
PD-1: programmed death-1
PMA: phorbol myristate acetate
PPARγ: peroxisome proliferator-activated-receptor gamma
PVN: paraventricular neuron
SCN, suprachiasmatic nucleus, SF-1: steroidogenic factor 1
SHP: small heterodimer partner
StAR: steroidogenic acute regulatory protein
STAT: signal transducer and activator of transcription
TCR: T cell receptor
TEC: thymic epithelial cell
TF: transcription factor
TGF: transforming growth factor
TJ: Tight junction
TNBS: 2,4,6-trinitrobenenesulphonic acid
TNF: tumor necrosis factor
TNFR: TNF-receptor
UC: ulcerative colitis.
